# Editorial

**Published:** 1867-07

**Authors:** 


### Editorial.

Illinois State Medical Society.—In the present number of the Examiner, will be found a full record of the proceedings of our State Medical Society, at its recent meeting in Springfield. There was a larger number of members in attendance than at any previous meeting for eight or ten years. It was characterized by harmony, strict attention to appropriate business, and a spirit of active investigation. The reports and papers presented were more numerous and interesting than usual, but the time allowed for their investigation was too short.

For the first time in several years, the Treasury was reported as not in debt, and money enough was received to ensure an early publication of the *Transactions* for 1867. We hope those members who presented reports or papers, which were referred to the Committee on Publication, and retained them in their own hands, will forward them to the Secretary without delay. By so doing, they will greatly facilitate an early publication.

---

Medical Education.—We are gratified to observe that the proposed revision of our system of medical education, as adopted by the Convention of Medical College Delegates, recently held at Cincinnati, is receiving the cordial sanction of nearly all our exchanges. The editor of the *Boston Medical and Surgical Journal*, in a recent number, strongly endorses the action of the convention, but expresses regret that the "Elements of the Natural Sciences" should have been stricken out of the Section relating to Preliminary Education. Had he looked a little closer, he would have been spared this regret; for he would have discovered that though the words were stricken out at one stage of the meeting, they were restored again before the close of the session. That Section, as it was finally adopted, unanimously, by the convention, is as follows:—

"That every student applying for matriculation in a medical college, shall be required to show, either by satisfactory certficate, or by a direct examination by a committee of the Faculty, that he possesses a thorough knowledge of the common English branches of education, including the first series of Mathematics and the elements of the Natural Sciences, and a sufficient knowledge of Greek and Latin to understand the technical terms of the profession; and that the certificates presented or the results of the examination thus required, be regularly filed as a part of the records of each medical college."

We hope to see the whole plan of revision, as recommended by the convention, *practically* adopted by the colleges and the profession in 1868.

---

The Quarterly Journal of Psychological Medicine and Medical Jurisprudence.—It is announced that a new periodical, with the above title, will be issued on the 1st of July, in the City of New York. It is to be edited by William A. Hammond, M.D.

---

Obituary.—The daily papers have just announced the death of Geo. K. Amerman, M.D., of this city. He died at Marcellus, New York, whither he had gone on account of failing health. Dr. Amerman was well known in this city as an active and intelligent member of the profession, and his early death will be universally regretted. He died from consumption, in the 35th year of his age.

ANNUAL ANNOUNCEMENT.—We have received the Ninth Annual Announcement of Chicago Medical College for the Lecture Term of 1867–8 As the announcement will reach our readers at the same time with this number of the EXAMINER, we need do no more than call attention to it. We hope every physician and student into whose hands it may fall will give it a careful perusal. It contains not one word of false pretension, but represents an institution which, for completeness in its system of instruction, the number of its faculty, and its clinical advantages, has no superior in this country.

---

CORRESPONDENTS.—We have received, too late for insertion in the present number, the proceedings of the Morgan County Medical Society, the Adams County Medical Society, and some other papers. They will appear in our next issue.

---

DR. BAKER BROWN OF LONDON.—The celebrated Dr. BAKER BROWN has recently been expelled from the Obstetrical Society of London, for unprofessional conduct, in connection with his operations of clitoridectomy for the cure of epilepsy.

---

WANTED.—Three copies of the *Transactions of the Illinois State Medical Society* for the years 1853 and 1855. These copies are wanted to complete the files kept for the use of the Society. Hence, any reader who may have one or both of the above numbers on hand, and does not wish to keep a full record, will confer a great favor by sending the same to the undersigned, Permanent Secretary of the Society.

N. S. DAVIS,

*166 State St., Chicago.*

Money Receipts from May 27th to June 26th.—Drs. B. Wilson, Chambersburg, $3; V. L. Hurlbut, Chicago, Ill., 3; P. S. Macdonald, Chicago, Ill., 3; J. T. Frazer, Howard's Point, Ill., 3; D. O. Crist, Bloomington, Ill., 3; C. R. Parke, Bloomington, Ill., 3; T. Nicols, Peshtigo, Ill., 3; H. L. Smith, Orland, Ind., 3; W. E. Peters, Nine Eagles, Iowa, 1 50.

---

Mortality Report for the Month of May:—

CAUSES OF DEATH.

| | | | |
|---|---|---|---|
| Abscess, | 1 | Epilepsy, | 1 |
| Accidents, | 11 | Erysipelas, | 1 |
| Apoplexy, | 2 | Fever, Typhoid, | 5 |
| Croup, | 3 | Fever, Lungs, | 6 |
| Cancer, | 3 | Fever, Scarlet, | 5 |
| Consumption, | 32 | Fever, Ship, | 1 |
| Cholera Morbus, | 1 | Fever, Typhus, | 2 |
| Convulsions, | 39 | Fever, Gall, | 1 |
| Canker Sore Mouth, | 1 | Fever, Nervous, | 1 |
| Congestive Chills, | 2 | Fever, Puerperal, | 1 |
| Congestion of Brain, | 2 | Fever, Congestive, | 1 |
| Congestion of Bowels, | 2 | Inflammation of Lungs, | 10 |
| Congestion of Lungs, | 2 | Inflammation of Brain, | 1 |
| Congestion, | 1 | Inflammation of Heart, | 1 |
| Childbirth, | 1 | Measles, | 3 |
| Colic, | 1 | Nephritis, | 1 |
| Diphtheria, | 7 | Old Age, | 5 |
| Drowned, | 6 | Phthisis, | 2 |
| Disease of Heart, | 5 | Poisoning, | 1 |
| Disease of Lungs, | 3 | Pneumonia, | 1 |
| Disease of Brain, | 1 | Small-Pox, | 3 |
| Disease of Liver, | 1 | Stillborn, | 5 |
| Debility, | 3 | Spasms, | 2 |
| Dropsy, | 7 | Suicide, | 1 |
| Diabetes, | 2 | Teething, | 5 |
| Dysentery, | 2 | Whooping-Cough, | 11 |
| Decline, | 1 | Unknown, | 21 |
| Delirium Tremens, | 1 | | |

Total, 241

Total number last year for the month of May, 275

Total number during the month of April, 278

Total number during the month of May, 241

Decrease, 37

DIVISIONS OF THE CITY.

North,........ 56 | South,........ 83 | West,.........111 | Total,.........240

Unknown, 1

Ages of the Deceased.—Under 5 years, 108; over 5 and under 10 years, 9; over 10 and under 20, 9; over 20 and under 28, 35; over 30 and under 40, 30; over 40 and under 50, 17; over 50 and under 60, 13; over 60 and under 70, 12; over 70 and under 80, 5; over 80 and under 90, 3; over 90 and under 100, 1; unknown, 6. Total, 241.

| | | | | | |
|---|---|---|---|---|---|
| Chicago, | 105 | France, | 1 | Sweden, | 6 |
| Austria, | 1 | Germany, | 25 | Scotland, | 2 |
| United States, | 40 | Ireland, | 40 | Unknown, | 2 |
| Canada, | 4 | Isle of Man, | 1 | | |
| England, | 7 | Norway, | 4 | Total, | 241 |

